# Defending an inclusive right to genital and bodily integrity for children

**DOI:** 10.1038/s41443-021-00503-x

**Published:** 2021-12-02

**Authors:** Kate Goldie Townsend

**Affiliations:** Department of Politics, University of Exeter, Exeter, United Kingdom

**Keywords:** Anatomy, Health care

At the time of writing in mid-2021, policy on child genital cutting and modification is inconsistent in the UK, US, and most European states, and there is growing consensus that this inconsistency should end [1–10]. The question addressed here, is whether Western liberal democracies ought to discourage, if not legally prohibit, all forms of medically unnecessary child genital cutting and modification, or permit some relatively minor forms. Given the core political values of Western liberal democracies, including a commitment to human rights, this piece takes a liberal normative approach and argues that individual rights to bodily – and especially genital – integrity should take priority over group rights *if* they come into conflict.

## Current inconsistencies in law and policy

The idea of the child’s right to bodily integrity has increasingly been defended in bioethical, philosophical, and legal scholarship [1–10]. Some authors argue in favour of the child’s right to genital integrity grounded in the value of genital and sexual autonomy for all individuals [6–8]. The aim is to protect all children, whatever their sex-trait characteristics or associated sex category assignment, and whatever the sociocultural preferences of their parents, from medically unnecessary^1^ genital cutting and modification practices until they reach an age of legal majority [6–11]. Once a person has become an adult and is deemed competent to make considered decisions about practices that involve surgical risk and typically permanently alter their sexual anatomy, the state should not seek to prevent them from pursuing such operations. The position I advocate is fairly simple: children should be protected from medically unnecessary genital cutting and/or modification until they are adults; once they become adults, they should be permitted to have their genitalia modified should they so choose.

Though simple, this position is at odds with most current policy in Western liberal societies [4, 7, 8, 10–14]. Children with female-typical sex characteristics in Western (and many other) societies are legally protected from medically unnecessary genital cutting and modification, however ‘minor’ the cutting may be, and even if sought by parents for religious reasons. Children with male-typical sex traits on the other hand, are not legally protected from genital cutting practices, even when the practice is considerably more physically invasive than some of the prohibited types affecting children with female-typical sex traits, for instance, symbolic nicking [7–11]. Children with intersex traits or differences of sex development also lack legal protection from medically unnecessary genital cutting and modification, even when the modification is *as* if not *more* physically invasive than prohibited procedures affecting children with female-typical sex characteristics [8, 15], with a few notable exceptions [8, 16].

Different treatment of child genital cutting practices depending on the sex of the child is morally problematic and could potentially be ruled unconstitutional in Western states [17]. It is indicative of cultural bias that bestows preferential treatment on the practices of Western majority and established minority groups, even when those practices are materially similar to the strictly prohibited practices of marginalised minority groups [4, 7, 8, 11–14]. This bias is evident in the genital cutting policies of international liberal institutions including the World Health Organisation (WHO) [18, 19], and it is transparent in current UK legislation on Female Genital Cutting (FGC). The Female Genital Mutilation Act 2003 [20] criminalises the cutting of adult female genitals for ‘cultural’ reasons even if the woman has made it clear that she wants to be cut or sewn [12, 20]. Genital modification practices are permitted if the modification is ‘necessary for her… mental health’ but ‘it is immaterial whether she or any other person believes that the operation is required as a matter of custom or ritual’ ([20], section 1). This means that in practice, the policy mainly affects women from marginalised cultural and ethnic groups whose cutting practices are understood to be ‘matter[s] of custom or ritual’, while (primarily white) majority group women can have their genitals modified if it is deemed important for their mental health [8, 12, 20].

Policy inconsistency of this kind, that infantilises women from marginalised groups (the UK law explicitly describes them as ‘girls’), is a hangover from a long trend of cultural supremacism in Western policy-making and political theorising [3, 5, 8, 12–14]. Policymakers who target groups practising medically unnecessary female child genital cutting (FGC) but remain silent over or even endorse medically unnecessary male and intersex child genital cutting (MGC, IGC) fail to consider how all child genital cutting practices are maintained and driven by ‘cultural’ norms [7, 8, 12–14, 18, 19]. Shweder’s [5] contribution to the debate about genital cutting is very important here; he challenged Western critics of FGC to ‘take a hard second look’ at the practice and to be slow to judge the people and groups for whom it remains important. It is an invitation to evaluate the cultural practices and inherent biases of one’s own sociocultural context and heritage that many scholars pursuing policy parity on child genital cutting and modification have taken seriously [4, 5]. Most scholars working on the ethico-legal status of child genital cutting in Western societies have come to agree on one thing: policy inconsistency such as strict prohibition of so-called ‘cultural’ FGC alongside legal permission of multiple forms of medically unnecessary MGC and IGC cannot be reconciled with a principle of policy parity for diverse groups, nor defended if equal children’s rights to bodily integrity are taken seriously. What remains to be agreed upon, however, is what Western states ought to *do* about their inconsistent policies.

## Pursuing policy parity

One way to ensure policy parity regarding child genital cutting would be to permit some forms of FGC that are currently illegal. Advocates of tolerance for what they regard as ‘minimal’ forms of child genital cutting (such as, nicking, pricking, or partial removal of the clitoral prepuce or hood, and/or cutting or excision of portions of the labia) argue as follows: parents are permitted to authorise medically unnecessary intersex child genital modification and male child prepuce removal (partial or total) in Western societies whatever their justification – and on the condition that all of the child’s parents agree in some societies, for instance in the UK [9]. As such, justice requires that parents also be permitted to authorise medically unnecessary FGC for their daughters, as practised, for example, within various Muslim communities [21], so long as the cutting is no more harmful than whatever is permitted for male children.

Defenders of this position characterise it as a ‘harm reduction’ approach, the idea being that permitting these relatively minor forms would dissuade community members from continuing more intrusive forms of FGC that carry a greater risk of resulting in lasting complications ([2]: p. 290). This position takes seriously the fact that some forms of FGC are more materially harmful than others, and that grouping all non-Western types under the provocative and demonising title ‘Female Genital Mutilation’, as is standard in Western law and policy, obscures these material differences [7, 8, 18–20]. In recent work, Duivenbode and Padela consider the question from a Muslim religious perspective, arguing that rather than ‘decoupling’ FGC from Islam, as is common for opponents of the practice, it would be beneficial to take guidance from Islamic ethical teachings that favour harm reduction [2]. The authors argue that, despite protestations to the contrary, there is a meaningful historico-religious association between Islam and FGC in many communities that should be acknowledged, rather than avoided, by people engaged in the debate ([2]: p. 290).

Presenting child FGC in this way may well be successful for its advocates. The argument that child MGC should be permitted has been most effective in real-world contexts when framed as a matter of religious freedom [8, 22–24]. For instance, in Germany, the decision to permit infant MGC for religious purposes was made on the basis that prohibiting it would be a violation of parents’ religious freedom [24]. Similarly, Iceland recently shied away from enforcing a ban on MGC after criticisms that it would violate the religious freedom of some practising groups [23]. The question of religious freedom is among the most difficult areas to tread politically speaking, and so ‘recoupling’ FGC with Islam is a potentially powerful way to argue that some forms should be permitted. However, the argument is unconvincing for several reasons.

First, the idea that male child prepuce removal is ‘harmless’ is highly controversial [1, 4, 7–9, 25, 26]. The suggestion that the practice should be used as a default standard for what is acceptable when considering harm-reduction approaches to FGC does not go unchallenged. Many authors have raised concern about the moral and legal status of male genital cutting practices, emphasising the material and psychological harms that they entail [1, 4, 7–9, 25, 26]. The view that penile prepuce removal is harmless assumes that the prepuce itself has no value, meaning that the only real harm at stake in its removal is the risk of surgical complications. But it is not standard to take this approach to other functional body tissues which are attributed their own value. The value given other body tissue means, for instance, that even when surgery is medically necessary, there is a moral and legal imperative to make every effort to preserve healthy tissue [27]. Many men whose genitals were cut as children, teens, or infants express extreme discontent at having their sexual anatomy altered before they were able to make the decision for themselves [25–28]. This does not mean, of course, that every person whose genitals were cut or modified in childhood experiences the same negative consequences, but it does cast serous doubt on the assertion that the harms of prepuce removal are minimal.

Second, from a practical point of view the argument has limited applicability to real-world cases because a great many justifications given for continuing child genital cutting practices simply are not religious. Routine secular child MGC in the US, for instance, is practised for various reasons, from parental aesthetic preferences to the medically controversial belief that prepuce removal promotes genital health or hygiene [1, 7, 8, 22, 24]. Intersex child genital modification is defended on the assumption that children have a psychosocial need for their genitals to fit a physical sex binary [8, 15, 16, 22]. Duivenbode argues that the blanket prohibition of medically unnecessary child genital cutting would further disadvantage marginalised minority religious groups [29], but importantly, religion is seldom the only justification cited to defend child genital cutting practices and is often not cited at all [30]. If child genital cutting and modification practices were prohibited out of respect for all children’s right to genital integrity whatever their sex characteristics, then not only religious groups would be affected. Parents and medics within dominant *and* marginalised groups in Western states would have their value preferences limited by prohibiting all child genital cutting and modification.

Finally, the underlying claim that collective rights to engage in other-affecting religious practices should take priority over the individual’s right to bodily integrity is hard to reconcile with the liberal commitments of Western states. While liberal thought and policy are increasingly open to group differentiated rights within culturally diverse societies, the individual remains normatively prior to the collective when the group’s freedom would upset the rights most prized by liberals; and bodily integrity is key for liberals of all stripes [1, 7–10]. Duivenbode argues that female and male child prepuce removal should be permitted in liberal democracies within an account of value pluralism whereby groups should be free to practise traditions that cohere with their internal value structures. Duivenbode is right to stress the importance of respect for all groups within democratic political societies, but for value pluralism to remain morally compatible with core liberal principles, there must be limits to what is permitted when it comes to other-affecting actions. Many children in democratic (and non-democratic) societies who are raised within religious value systems grow up to reject certain aspects of those systems and may seek to leave the group [31]. According to Möller, religious freedom properly understood, ought to include the possibility for those raised within a religious household to ‘distance’ themselves from that religion ([32]: p. 470). This ‘freedom restraint’ on what parents may legitimately do to their children:

‘prohibits irreversible religiously or culturally motivated changes to the child’s body: precisely by virtue of being irreversible, such changes make it impossible for the child to ever to [dissociate] from them and to live … life free from a religiously or culturally imposed physical mark’. ([32]: p. 470)

Möller’s argument here is that the physical mark means that the child will be permanently included in the group even if they come to reject its values and practices. It goes without saying that the child could still reject many of their parents’ religious teachings upon becoming an adult, and maybe endorse different religious teachings or become an atheist [32]. But, Möller points out, the physical mark would remain, and they may feel that their genital and bodily integrity had been unjustifiably violated before they could autonomously endorse or reject the associated values and practices [32].

Priority rules are necessary within value plural political societies to avoid slipping into a political space that permits people to do anything to anybody on the grounds that the action is important for their collective conception of the good life. When it comes to liberal societies, the individual’s right to bodily integrity must be prior to the group’s collective identity. Children’s rights to bodily and genital integrity function as liberty-limiting principles *if* they come into conflict with parental preferences that would violate those rights. The role of liberty-limiting principles in value plural liberal societies is to emphasise the limits of moral relativism, and to stress priority rules that constrain the practices of *all* groups – majority, minority, dominant, and marginalised.

## The right to bodily, including genital, integrity

The idea that people have a right to bodily integrity is commonplace and ‘now universally accepted’ ([33]: p. 569). It is enshrined in human rights law representing political and institutional commitments to respecting people’s bodies as sites of their ‘personal freedom’ [7–9]. The right consolidates, politically, a moral commitment to respecting the body as the point at which the moral person encounters the empirical world. The moral and political significance of the individual right to bodily and genital integrity concerns the profoundly personal value that bodies and genitals have for individuals’ flourishing and experience throughout life. Our^2^ bodies are crucial to the most important events of our lives, ‘being born, growing up, making love, having children, falling ill and dying’ ([34]: p. 1). We use our bodies to express our thoughts and feelings, to engage with the objective world, to hide from the social world. In interfering with my body, you interfere with my subjectivity in the most immediate, direct, and intimate way. Violations of bodily integrity, then, are violations of a most serious kind within a liberal normative framework.

A distinction: bodily *integrity* is complicated with, but distinct from and irreducible to bodily *autonomy* [8, 30, 33, 34]. A commitment to the principle of bodily integrity requires others to respect individuals’ bodies, to leave them uncoerced, unpenetrated, and uncut whether or not the individual is *autonomous*. The normative thrust of a commitment to bodily integrity resides in the value and significance of the body itself as commanding respectful treatment by others – this value and significance is present whether or not the person is autonomous and capable of consenting to interventions into their body. This matters conceptually and with regards to the argument against medically unnecessary child genital cutting, because it means that the right to bodily integrity is not merely about ensuring that the person is able to exercise rational control over their body, and it acknowledges the fact that individuals are seldom in complete control of their bodies. Understanding bodily integrity as distinct from bodily autonomy appeals to and accounts for the normative significance of the body itself as the point at which a person’s integrated subjectivity – in all its rational and irrational components – encounters the empirical world. Bodily integrity as the ‘integrated body’ helps ‘to explain the legal structure of the right, the normative weight of the right, and the ambiguous boundaries of the right’ ([33]: p. 567).

A body-oriented approach to understanding the right to bodily integrity attributes to the body a value of its own as a site of ‘moral experience’ ([30]: p. 188), its moral value is distinct from the person or people who exercise(s) control over it and it ‘cannot (entirely) be owned or controlled’ ([30]: p. 183). When it comes to the child, the right to bodily integrity has the character of protecting their *interest* in having an intact body, so long as there is not a medical need to interfere with the body, and carries with it a duty in others to respect their bodies as the physical boundary of their integrated subjectivity, and importantly, it emphasises the idea that the individual’s body cannot and should not be owned or controlled by others.

Dekkers et al. [30] identify a paradox in the moral outlook of religious groups that are committed to the concept of bodily integrity, but practise child genital cutting for religious purposes. The authors found that there are different views of bodily integrity, some of which contain the idea that child genital cutting is permissible because it is thought of as contributing to male children’s bodily integrity: without it, their bodies are viewed as imperfect. In their analysis of different perspectives on MGC and FGC of minors amongst people from Muslim and Jewish communities, they observe that while many of the people they interviewed did not consider bodily integrity to be violated by MGC, they invariably considered FGC to be a violation of bodily integrity ([30]: p. 188). The interviewees also reported a feeling of unease and discomfort when witnessing MGC, despite the fact that in their view ‘it definitely needs to be done’ (p. 188). Dekkers et al. claim that:

‘[t]his fact underscores that, although they rationally do not speak in terms of bodily integrity or of a violation of the human body, they intuitively express feelings of ambivalence and hesitation that can be explained in terms of respect for the integrity of the body’. ([30]: p. 188)

This sense of unease is attributable to the moral value of the body itself.

Dekkers et al. [30] acknowledge the difficulty in capturing the moral significance of bodily integrity, and gesture towards the fact that the concept is deployed and interpreted in different ways in many real-world contexts. Nonetheless, the following is profoundly important for how the right is conceptualised and deployed: the right to bodily integrity is not reducible to bodily autonomy, that is, a violation of the right to bodily integrity is not *only* a violation of a person’s autonomy. This means that a person who is not autonomous can have their bodily integrity violated. All of this matters here, because it means that the child’s rights to bodily and genital integrity are grounded in their interest in having their bodily integrity respected irrespective of whether they would or would not retrospectively endorse any cutting or modification of their genitalia as adults. It may well be the case that some adults who had their genitals cut or modified in childhood would affirm it as something they are content with because it coheres with the wider sociocultural values of their group (majority or minority, dominant or marginalised); but the practice would still be a violation of their bodily and genital integrity and simply cannot be justified by appealing to group rights to religious freedom within a liberal political framework.

## Footnotes

1 The Brussels Collaboration on Bodily Integrity [10] defines ‘medically necessary’ in the following way: ‘(1) the bodily state poses a serious, time-sensitive threat to the person’s well-being, typically due to a functional impairment in an associated somatic process, and (2) the intervention, as performed without delay, is the least harmful feasible means of changing the bodily state to one that alleviates the threat’.

2 By ‘our’, ‘we’, and ‘us’, I mean all embodied people.

## References

[1] Chambers C. Reasonable disagreement and the neutralist dilemma: abortion and circumcision in Matthew Kramer’s *Liberalism with Excellence*. Am J Jurisprud. 2018;63:9–32.

[2] Duivenbode R, Padela AI. The problem of female genital cutting: bridging secular and islamic bioethical perspectives. Perspect Biol Med. 2019;62:273–300. 31281122 10.1353/pbm.2019.0014

[3] Dustin M. Female genital cutting/mutilation in the UK: challenging the inconsistencies. Eur J Women’s Stud. 2010;17:7–23.

[4] Earp BD. Zero tolerance for genital mutilation: a review of moral justifications. Curr Sex Health Rep. 2020;12:276–88.

[5] Shweder RA. What about “Female Genital Mutilation”? And why understanding culture matters in the first place. Daedalus. 2000;129:209–32.

[6] Svoboda JS. Genital integrity and gender equity. In: Denniston GC, Gallo PG, Hodges FM, Milos MF, Viviani F (eds). Bodily integrity and the politics of circumcision. Dordrecht: Springer; 2006. pp. 149–64.

[7] Townsend KG. The child’s right to genital integrity. Philos Soc Criticism. 2020;46:878–98.

[8] Townsend KG. The child’s right to genital integrity: protecting the child, rejecting harmful practices, and enabling sexual autonomy. PhD dissertation. Open Research, Exeter: University of Exeter; 2021.

[9] Möller K. Male and female genital cutting: between the best interest of the child and genital mutilation. Oxford J Legal Stud. 2020:1–25.

[10] Brussels Collaboration on Bodily Integrity. Medically unnecessary genital cutting and the rights of the child: moving toward consensus. Am J Bioeth. 2019;19:17–28. 10.1080/15265161.2019.1643945 31557092

[11] Earp BD. The child’s right to bodily integrity. In: Edmonds D, (ed). Ethics and the Contemporary World. Abingdon and New York: Routledge; 2019. p. 217–35.

[12] Shahvisi A, Earp BD. The law and ethics of female genital cutting. In: Creighton S, Liao LM, (eds). Female genital cosmetic surgery: solution to what problem? Cambridge: Cambridge University Press; 2019. p. 58–71.

[13] Galeotti AE. Relativism, universalism, and applied ethics: the case of female circumcision. Constellations. 2007;14:91–111.

[14] Galeotti AE. Autonomy and cultural practices: the risk of double standards. Eur J Political Theory. 2015;14:277–96.

[15] Ehrenreich N, Barr M. Intersex surgery, female genital cutting, and the selective condemnation of “cultural practices”. Harvard Civil Rights-Civil Liberties Law Review. 2005: 114–20. https://papers.ssrn.com/sol3/papers.cfm?abstract_id=2926589.

[16] Monro S, Carpenter M, Crocetti D, Davis G, Garland F, Griffiths D, et al. Intersex: cultural and social perspectives. Cult Health Sex. 2021;23:431–40. 33783329 10.1080/13691058.2021.1899529

[17] Earp BD. Why was the US ban on ‘female genital mutilation ruled unconstitutional, and what does this have to do with male circumcision? Ethics Med public health. 2020;15:100533.

[18] The World Health Organisation. Eliminating female genital mutilation. Geneva: WHO Press; 2008.

[19] The World Health Organisation. Neonatal and male child circumcision. Geneva: WHO Press; 2010.

[20] Female Genital Mutilation Act 2003. UK: The Stationary Office Limited. https://www.legislation.gov.uk/ukpga/2003/31/pdfs/ukpga_20030031_en.pdf.

[21] Bootwala. A review of female genital cutting (FGC) in the Dawoodi Bohra community: parts 1, 2, and 3. Curr Sex Health Rep. 2019;11:212–35.

[22] Earp BD, Johnsdotter S. Current critiques of the WHO policy on female genital mutilation. IJIR: Your Sex Med J. 2021;33:196–209. 10.1038/s41443-020-0302-0 32457498

[23] Sherwood H. Iceland law to outlaw male circumcision sparks row over religious freedom. The Guardian. 2018. https://www.theguardian.com/society/2018/feb/18/iceland-ban-male-circumcision-first-european-country.

[24] Merkle R, Putzke H. After Cologne: male circumcision and the law. Parental right, religious liberty or criminal assault? J Med Ethics. 2013;39:444–9. 23698890 10.1136/medethics-2012-101284

[25] Earp, B, & Darby, R (2017). Circumcision, sexual experience, and harm. Univ Pa J Int Law. 2017;37:1–56.

[26] Hammond T, Carmack A. Long-term adverse outcomes from neonatal circumcision reported in a survey of 1,008 men: an overview of health and human rights implications. Int J Hum Rights. 2017;2:189–218.

[27] Darby R. Risks, benefits, complications and harms: neglected factors in the current debate on non-therapeutic circumcision. Kennedy Inst Ethics J. 2015;25:1–34. 25843118 10.1353/ken.2015.0004

[28] Remennick L. Joining the tribe: adult circumcision among immigrant men in Israel and its traumatic aftermath. Cult Health Sex. 2021:1–27. 10.1080/13691058.2021.1879272. 10.1080/13691058.2021.1879272 33512277

[29] Criminalizing medically unnecessary child genital cutting in Western countries: the terms of the debate and some reasons for caution. Int J Impot Res 2021. 10.1038/s41443-021-00491-y. 10.1038/s41443-021-00491-y 34799711

[30] Dekkers W, Hoffer C, Wils JP. Bodily integrity and male and female circumcision. Med, Health Care Philos. 2005;8:179–91. 16215798 10.1007/s11019-004-3530-z

[31] Mills C. The child’s right to an open future? J Soc Philos. 2003;34:499–509.

[32] Möller K. Ritual male circumcision and parental authority. Jurisprudence. 2017;8:461–79.

[33] Herring J, Wall J. The nature and significance of the right to bodily integrity. Camb Law J. 2017;76:566–88.

[34] Wicks E. The State and the Body: legal regulations of bodily autonomy. Oxford: Hart Publishing; 2016.

