# Correction: Defending an inclusive right to genital and bodily integrity for children

**DOI:** 10.1038/s41443-022-00545-9

**Published:** 2022-03-04

**Authors:** Kate Goldie Townsend

**Affiliations:** Department of Politics, University of Exeter, Exeter, UK

**Keywords:** Anatomy, Health care

Correction to: *International Journal of Impotence Research* 10.1038/s41443-021-00503-x, published online 2 December 2021

For this article parts of the text have been converted to footnotes. They read as follows:

The Brussels Collaboration on Bodily Integrity [10] defines ‘medically necessary’ in the following way: ‘(1) the bodily state poses a serious, time-sensitive threat to the person’s well-being, typically due to a functional impairment in an associated somatic process, and (2) the intervention, as performed without delay, is the least harmful feasible means of changing the bodily state to one that alleviates the threat’.

By ‘our’, ‘we’, and ‘us’, I mean all embodied people.

